# What happens when a country bleeds soft power? Conceptualising ‘Negative Watch’: towards an epistemology for negative and adversarial place branding

**DOI:** 10.1057/s41254-023-00302-9

**Published:** 2023-05-26

**Authors:** Sameera Durrani

**Affiliations:** University of Technology Sydney, Sydney, Australia

**Keywords:** Public diplomacy, Semiotics, Soft power, Iran, Pakistan, Place branding, International relations, Nation brands

## Abstract

This article contends that the classic public diplomacy model prioritising scholarly research on maximising soft power in the eyes of foreign publics needs to be extended. It proposes a new concept, Negative Watch, which dyadically complements Nye’s in (Ann Am Acad Polit Soc Sci 616: 94–109, 2008) idea of soft power. By extending the epistemology surrounding negative place branding, this paper seeks to expand the debates around it. Where soft power is about accruing power via credibility, Negative Watch maps its loss, approaching it from a communicative dimension. Using a case study approach, the article presents the concept as a model with two key components (1) An index comprising a typology of unfavourable narratives connoting negative affect towards nation brands; (2) a heuristic model outlining a spectrum of outcomes precipitated by reputational decline. The index can be used as a self-assessment tool by a nation to track its reputational erosion, so that it may take measures before it progresses too far down the spectrum of consequences. It can also facilitate systemic assessment of external actors, including allies as well as adversaries, to facilitate strategic decision making within the realm of international relations and public diplomacy.

## Introduction

*“I was born in Kurdistan. My city was bombed for 8 years….so all I hear [is] the noise of sirens, the noise of guns, the noise of machine guns …. So, I don’t want to live in America, because I don’t want to hear the noise of guns. I don’t believe that guns bring peace and tranquillity for me”.*

Moz Azimitabar, Kurdish Refugee, on why he won’t seek asylum in the United States (The Project, 2022).

National reputations, national brands, matter. We decide how to think and feel about a country based on its reputation. If we have never been there, what we see in media narratives plays a big role in shaping these perceptions (Dolea 2015). These reputational representations collectively influence and shape global behaviours towards a country. Critically, though, these representations are not necessarily stable. The United States has long depicted itself as ‘the land of the free’. To Mr. Azimitabar, in 2022, the United States is a violent land of guns—a reflection on the increasing precarity of global soft power dynamics. The rise of populism, global socio-economic uncertainty, and prevalent social media dynamics have complicated the management of national reputations. This calls for a review of the lexicon of public diplomacy and place branding, to better describe, diagnose, understand, and predict these new trends.

Presently, public diplomacy and place branding emphasise epistemology and frameworks examining the acquisition of credible, attractive, and national reputations. The classic public diplomacy model foregrounds favourable images of a country’s policies, actions, and systems (Gilboa 1998; Wang 2006) epitomised by Joseph Nye’s (2008) theory of soft power. Nation branding, affiliated with marketing, focusses on enhancing a nation’s competitive identity (Anholt 1998, 2005, 2007). There is a need, therefore, to craft theoretical frameworks examining the reverse: *the process whereby states lose soft power*, *and what happens when they do.* This article aims to address this gap. Building on Nye’s (2008) conceptualisation of soft power, it proposes a new theory: ‘Negative Watch’. The phrase itself is inspired by investment terminology, where it is defined as a status assigned by credit rating agencies to companies when lowering their credit ratings. It is an indication that a company’s ability to repay its debts may be deteriorating (Chen 2021). While acknowledging that global political power dynamics operate in a differently nuanced and complex fashion as compared to the financial sector—there is no one visible or invisible hand that can unilaterally raise or lower a country’s reputational security ratings—this paper extrapolates the central conceptual premises of credibility loss and curtailed agency, appropriating and contextually operationalising this term for the domain of nation branding and public diplomacy. It argues that a nation consistently losing reputational credits, and therefore, soft power, earns a place on a reputational ‘Negative Watch’, with a consequent impact on how it is treated by others in the global political arena.

Soft power—a complex idea extending across many actors and institutions—has been critiqued for conceptual ambiguity and over-reach (Fan 2007; Mattern 2005). Therefore, this initial conceptualisation of ‘Negative Watch’ focusses on the communicative aspect of soft power loss, given the centrality of communication flows to mediating national reputations. As Joseph Nye (2010a, b) notes, “It’s not whose army wins. It’s also whose story wins. And we have to think more about narratives, and whose narrative is going to be more effective”. Storytelling is central to mediation of soft power gain and loss—hence, it is the designated focus for the proposed framework.

Furthermore, as noted by Cull (2022), the contemporary global political landscape necessitates a more nuanced understanding of soft power dynamics, along a continuum mapping its presence *and* absence, gain *and* loss. Soft power, from its first articulation at the end of Cold War, has served as the core idea underpinning the public elements of diplomacy around the world. For the post-Cold War world, it was the perfect idea for understanding an international system looking to understand how the West did so well, and Soviet Russia, so poorly. This has changed. Cull (2022a) argues that the complexity of a politically multi-polar post-pandemic world necessitates a dual focus on reputation and collaboration, whereby a country’s ‘reputational security’ is conceptualised as being reliant on reputational assets *and* vulnerabilities. Within public diplomacy, as explored in the following section, a strong, existing tradition of research examining reputational assets, centred around Nye’s epistemology around soft power, already exists. Building on Cull’s (2022) argument, this paper proposes a new theory, which conceptualises the second half of dyad comprising reputational security—the absence of soft power, and outlines terminology for making it visible to academic and policy audiences in public diplomacy.

The theory is devised using a constructivist, interdisciplinary approach, drawing on communication, journalism, and public diplomacy, following existing precedents for using interdisciplinary, methodological, theoretical approaches in public diplomacy (Sevin et al. 2019). To this end, it draws on a longitudinal content analysis of the media coverage of two case studies with established reputational challenges across a period of thirty years: Iran and Pakistan. The archival data are purposively sampled from the visual coverage of an elite, historical global publication, *Time* magazine, specifically, its Asia Edition, given the primary case studies belong to that region. While pan regional content can vary across various editions of the magazine, the global content remains the same (Rohn 2010), as does the editorial philosophy, which usually resonates with prevalent western views and official U.S. policy, as noted by prior research using different editions (Griffin and Lee 1995; Khan 2002).

In doing so, the study draws on a strong, established tradition in media studies examining the impact of photojournalism in providing ideological support and justification for the way a country or certain communities are othered, particularly in the context of armed conflicts. As Payne (2012) argues, photography has been used for a long time to map territories, justify invasions, and promote colonial settlement. Research has examined the existence of this trend across multiple international conflicts, including World War II (Engle 2012; Flamiano 2010); the 1992, 2003 Gulf Wars and the invasion of Afghanistan (Griffin and Lee 1995; Fahmy 2004 Griffin 2004; King and Lester 2004; Klaus and Kessal 2005; Parry 2011); the Syrian conflict (Mast and Hanegreefs 2015), and so on. While the proliferation of citizen photojournalism on social media has changed and complicated the visual mediatisation of armed conflicts (Mortensen 2015), visuals remain a key source for articulating national images, and influencing perception and policy towards a country, and as such, represent a highly relevant data set for examining the role played by representational processes in diminishing national reputations.

It provides the basis for a heuristic typology of negative reputational narratives, derived inductively using a grounded theory approach, whereby theory is ‘grounded’ in patterns generated from systematically collected data (Charmaz 2003), following existing traditions in public diplomacy for theorising inductively (Entman 2008; Gilboa 1998). The validity of these patterns is further explicated using other contemporary examples, with a focus on the United States, a country that has both deployed, and been subjected to, elements of Negative Watch, and has, post pandemic, experienced a decline in its reputational standing relative to other nation states (Cull 2022).

This is a methodological strength, as well as a limitation. The focus on a single source of data, and two primary case studies, enables a focussed analysis of generational, archetypical, consistent negative place branding narratives. However, as indices in the field of place image often use multiple surveys, focus groups, and proxy indicators, it is emphasised here that this qualitatively, inductively derived model is presented here as an initial, suggested epistemological structure for future debates about reputational loss, as was true of Nye’s (2008) initial conceptualisations of soft power, for scholars in the field to debate, strengthen, and expand contextually, using other methods. The aim of the case study analysis here is to conceptualise and illustrate the significance of establishing measures for tracking national reputational loss and its consequences.

To this end, this paper first situates the concepts by presenting an overview of soft power and its critiques, linking it to a definition of Negative Watch. This is followed by an overview of the methodology, and a synopsis of reputational challenges faced by chosen case studies. Then, it outlines the Negative Watch Index (NWI), illustrated with relevant examples, as well as a heuristic model of the spectrum of outcomes for being placed on Negative Watch. Finally, it discussed the concept’s strategic significance, limitations, and future applications.

## Soft power—a conceptual overview

This paper introduces a new concept to fill in a gap in the current foundational theory underpinning the idea of soft power. To this end, this section first situates the concept broadly within nation branding. It then outlines and critiques Nye’s (2008) conceptualisation of soft power, thereby demonstrating the existing gap to be addressed.

Nation Branding is categorised using three paradigms: technical–economic, which explores how nations compete in the global marketplace; the political approach, which focusses on public diplomacy perspectives, including Joseph Nye’s work; and critical/cultural approaches, which include historical and political economic analyses (Kaneva 2011). This project draws inspiration from all three. It builds on critical/constructivist critiques of Nye’s (2008) political soft power theory, to lay the grounds for a diagnostic narrative index following the technical–economic approach, derived using historical analysis.

The foundational concept here is soft power. In 1990, Joseph Nye introduced the term ‘soft power, arguing that a changing global environment called for less reliance on conventional military power, and greater exploration of co-optive power (ideology, cultural resources, etc.). Presciently, he predicted that the information revolution rendered persuasion strategically critical (Nye 2002). Defining it as the ability to get what you want through *attraction*, rather than coercion or payments, Nye identifies soft power as comprising *culture* (when it is pleasing to others), *values* (when they are attractive and consistent) and *policies* (Nye 2008).

Over the years, Joseph Nye has written extensively, and influentially, on various dimensions of soft power. He critiques the U.S trend of defunding cultural and educational exchanges with developing countries, noting China’s increased investment in the area (Nye 2007). He emphasises the importance of collaborating with NGOs, corporate actors, and using military officer exchanges and joint training as key strategies (Nye 2008). He critiques the post-9/11 coercive mode of American public diplomacy as undercutting American soft power efforts (Nye 2009), while noting the rise of China’s soft power (Nye and Wang 2009; Nye 2010b). He also coined the term ‘smart power’, which advocates a combination of hard and soft power resources (Nye 2011), and ‘sharp power’ (Nye 2021), which pierces political and information environments in target countries, and differs from soft power, which relies on openness with limits on deliberate deception.

While incredibly influential, Nye’s work has attracted criticism. Lukes (2007) sees soft power as a blunt instrument failing to distinguish between nuances of persuasion. Nye (2021) has responded by relating soft power to the third dimension of Lukes’s (2005) conceptualisation of the three faces of power (decision making, agenda setting, thought control). Roselle, Miskimmon, and Loughlin (2014) note how measuring and tracing the impact of soft power is problematic, instead preferring ‘strategic narratives’, e.g. Mohammed Khatami’s ‘Dialogue among Civilizations’.

Scholars from the constructivist paradigm critique Nye for ignoring the affective component of soft power and defining the concept of attraction in a ‘fuzzy’, confusing way. This is significant oversight, as attraction is central to the idea of soft power, and affective investment is key to establishing attraction (Solomon 2014). These scholars ask—what is attraction, how can it be measured better, and what is the role of emotion in regulating attraction?

Constructivist scholars argue that soft power drives from cultural and ideological attraction, and as such, is informed by affect and contingent cognitive framing (Mattern 2005; Solomon 2014; Haugaard 2019). As Mattern (2005) notes, actors can evoke desired affective responses from their target audience by using different types of sociolinguistic constructions of reality. Drawing on this critical, constructivist tradition, this paper argues that if gaining soft power, a narrative phenomenon contingent on positive affective assessment, is a matter of creating attractive sociolinguistic constructions of reality, then its loss can be facilitated by narratives evoking *repulsion* via negative affective associations. It is useful to track *both* attraction and repulsion; as Rothman (2011) notes, soft power resources can impact outcomes by making one alternative more attractive than another. There is growing recognition of the importance of analysing mechanisms applied to diminish national image and reputations of adversary states—Gilboa (2015), for instances, uses the term ‘brandjacking’ to describe the mechanisms underpinning strategies for actively diminishing a rival state’s reputation and image. Leveraging inspiration from these political and technical–economic perspectives (Nye 2008; Anholt 2007), this article outlines the conceptual mechanisms and implications underlying the sociolinguistic construction of repulsion towards a nation brand. The following section outlines key terms.

## Conceptualising Negative Watch

For the purposes of this paper, Negative Watch is conceptualised as a theory of public diplomacy and place branding examining variables negatively affecting national reputational credit reserves. It functions analytically as a complementary term for soft power. As Cull (2022a) notes, reputational security is mediated by soft power gain *and* loss, and therefore, policy makers and scholars in public diplomacy and place branding would benefit from epistemology examining the latter, as well as the former.

Therefore, the paper operationalises the theory of Negative Watch as a model comprising two sub-elements. First, an index that provides a measure of negative representational effects. Second, a heuristic model that outlines the significance of tracking these effects by illustrating adverse security outcomes of reputational collapse. The aim here is to broaden our understanding of soft power in a way that addresses and broadens existing debates and critiques around it.

It is important to acknowledge here that the gain or loss of soft power is a complex phenomenon involving many different factors, actors, and institutions (Fan 2007; Kassab 2020). A comprehensive debate on all these is beyond the scope of a single paper. Nye’s conceptualisations of soft power have been critiqued for ambiguity (Mattern 2005) and conceptual over-reach (Fan 2007), with soft power seen to ‘mean almost everything and therefore almost nothing’ (Hoagland 2004). Therefore, for clarity, the paper limits itself to a constructivist view of how power loss is constructed and facilitated using media narratives. As is true of Nye’s work, future scholarship may build on this epistemology by interpolating other factors contributing to soft power loss.

For the purposes of this paper, Negative Watch is operationally defined here, therefore, as the process whereby a consistent set of unfavourable media narratives contribute to the erosion of a country’s political legitimacy, credibility, and soft power. Building on Nye (2008), this paper argues that for a nation state, loss of soft power inevitably leads to lowered political credibility and legitimacy. Credibility is key to soft power, and politics is a contest of ‘competitive credibility’ (Nye 2008, p.100). Diminished credibility leads to lowered political legitimacy in the eyes of foreign governments and publics; hence, declining soft power. Over time, this results in a curtailed ability to exercise global, political influence, coupled with an increased vulnerability in terms of the way other actors exercise power over the country. This paper argues that a nation consistently losing soft power, earns a place on ‘Negative Watch’.

As outlined above, the theory is explained via two dimensions relevant to nations both as a way of viewing themselves and other actors: (1) An index that measures the applicability of the Negative Watch model to a country, (2) a heuristic model outlining a spectrum of outcomes of being on negative watch. The index, when used as a self-assessment tool, enables a nation to track its reputational erosion and take measures before it progresses too far down the spectrum of consequences. It can also facilitate systemic assessment of external actors, including allies as well as adversaries, to gauge their reputational security status (Cull 2022) to guide strategic decision making. While conceptualised qualitatively here, the indices, and the heuristic model, can be potentially quantified using scaled interval data scores (say, performance on each sub-variable could be scored using a numbered scale e.g. 1–10).

**Fig. 1:**
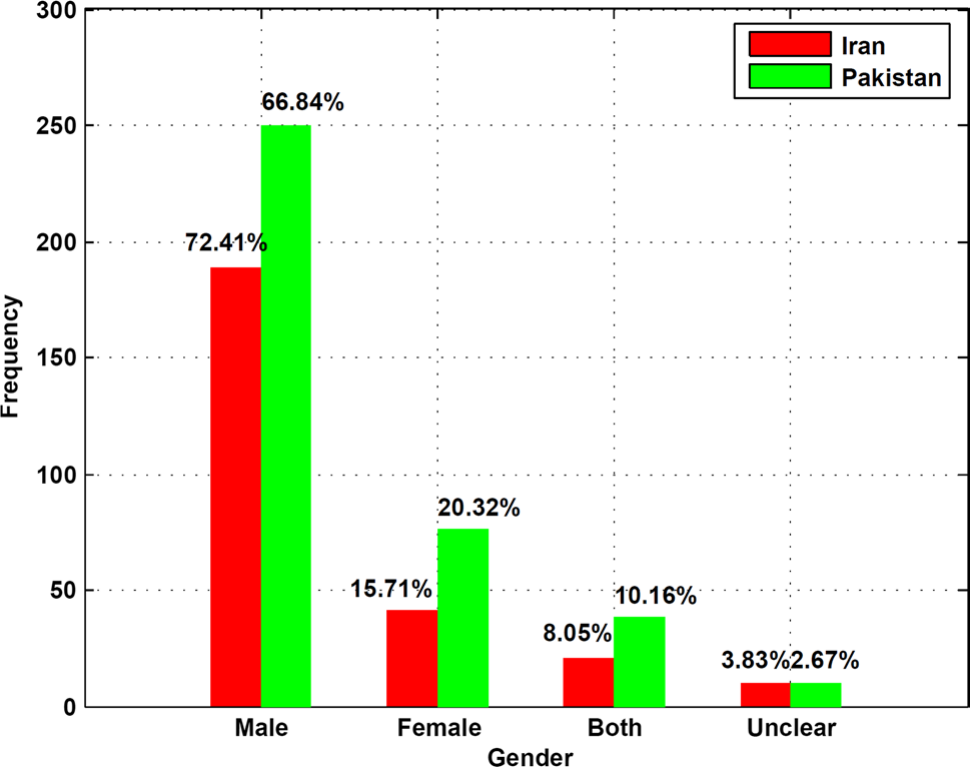
Representational Analysis: Gender (1981–2010)

**Fig. 2:**
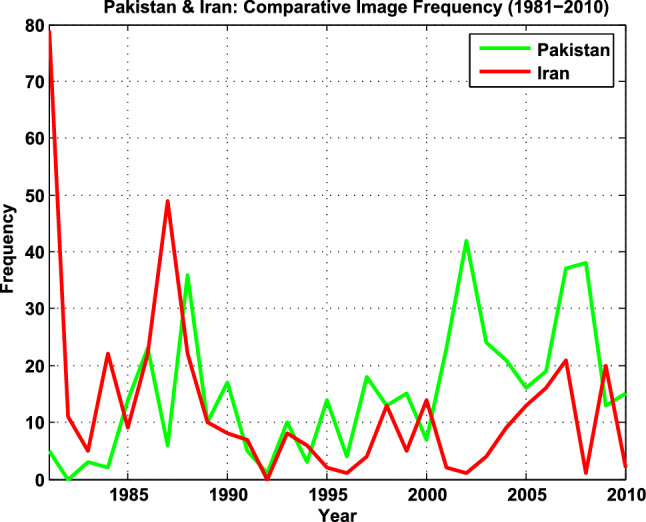
Pakistan and Iran—A Timeline of Comparative Image Frequency

It is worth noting here that efficacy of Negative Watch relies on four components—the *‘watcher’,* the *‘watched’,* the *platform,* and the *audience*. The final element, audience, is a particularly crucial one. The efficacy of all six elements of Anholt’s Nation Brand Index, for instance, is reliant on their relevance to audiences to translate into benefit. The same is true of Negative Watch. Certain audiences may disapprove of autocratic governance modes. Others may not. Some audiences may be repelled by a heavy handed approached towards political dissent. Others may not. These attitudes will in turn be mediated and strengthened by how they perceive the watcher, the affordances of the platforms from which they receive their information, and existing perceptual biases around the ‘watched’ entity. The complexities of these dynamics are noted here to illustrate the methodological challenges of studying soft power gain and loss—and therefore, it is useful again to acknowledge that while this paper aims to provide a conceptual basis for *initiating* debates around the discursive gap of how soft power is lost, it does not claim to be the final word. Future scholarship examining negative place branding from an audience studies perspective, analysing the impact of visual imagery in provoking affect amongst publics (Dolea 2022), would comprise a valuable addition to the field.

For the two historical case studies included here, the ‘watched’ entities are Pakistan and Iran. The ‘watcher’ is America, represented by data from *Time,* Asia–Pacific edition, (platform) whose audiences are elite publics in Asia Pacific. These elements will change with each case study. For instance, America is a ‘watched’ entity routinely critiqued by Rossiya Segodnya (Russia Today), a platform seen as representing Russia as a ‘watcher’ (Pisnia 2017), for audiences in various regions.

## Methodology

Using a grounded theory approach, and a combination of qualitative and quantitative data, the model is inductively derived and demonstrated via the analysis of three primary case studies: Pakistan, Iran, and the United States. This section outlines the methodology used for analysis, while the next one unpacks the patterns of reputational erosion characterising the chosen case studies, setting the stage for the delineation of the proposed model.

The historical case study data have been obtained from a longitudinal, qualitative content analysis (Mayring 2000) of a purposive sample of 840 images (Iran: 376 images, Pakistan: 454 images), from *Time* over 30 years (1 January 1981–31 December 2010). During this time period, Iran transitioned from the Islamic Revolution to the anti-orthodoxy Green Movement. Pakistan transitioned from the Afghan Jihad to the War on Terror, with the heroic mujahideen transformed into the feared Taleban. The chosen time period therefore contains rich data about national identity and image debates. Visual data are used, as it provides a powerful historical synopsis of the story of a nation brand. To paraphrase Baudry & Williams (1974–75), the ideological effects of the news photography apparatus may be disrupted by viewing it in a manner contrary to its conventions. Instead of synchronically examining individual photographs, this project looks at them altogether, much as one looks at unfolding individual frames in a film reel: components of the larger story or narrative, reflecting generationally reinforced, negative strategic narratives. The sample source, *Time,* emphasises visual storytelling, has a long publication history, and a prestigious status as an intermedia agenda setter (Vliengarth and Walgrave 2008). This makes it a suitable choice for a longitudinal content analysis of visual politics. The purposive sample (Sarantakos 1998, p.152) draws on relevant material from recurrent, visual sections of *Time:* Person of the Year, Best photographs of the year, photo features, photo essays, interviews, and feature articles.

**Fig. 3:**
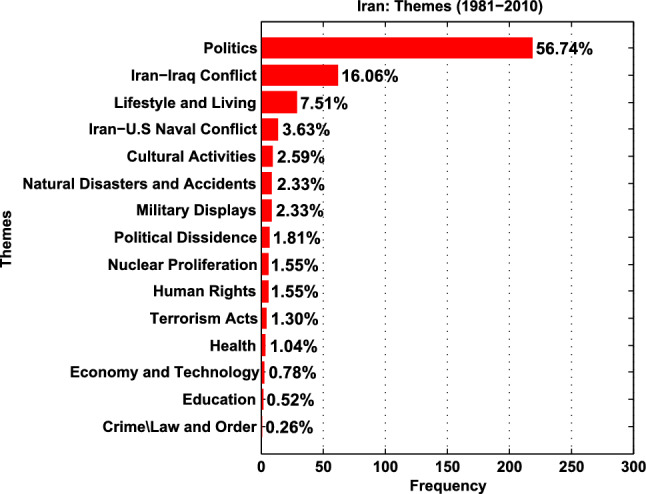
Iran—thematic portrayal

Social semiotic theory is a useful tool for ‘highlighting the crucial role visual images play in building, naturalizing, and reinforcing ideological messages’ (Ahmadgoli and Yazdanjoo 2019, 5). Building on this notion, the category system measures trends using two holistic semiotic categories:

- **Representation:** Representation, or the ideational metafunction, refers to ‘the ability of semiotic systems to represent objects and their relations in a world outside the representational system or in the semiotic systems of a culture’ (Kress and van Leeuwen 2006, p.47). This involves an analysis of the people represented in the pictures and the activities they are engaged in. This semiotic metafunction was operationalised for this study using two key sub-dimensions:
- **Thematic Portrayal** (sub-divided into a set of dominant narrative themes). The results for these are summarised in Figs. 2 and 3. For a detailed coding sheet, please see: https://osf.io/pvxak.
- **Key Actors** (the subjects of the photos, also referred to as represented participants). These were quantitatively coded with reference to age groups, nationality, and gender. Key sub-dimensions include (i) *Participant Status* (further sub-divided into elite actors. i.e. individuals identified by name in the caption/headline, from various professions such as politics, sports, human rights, who are well known within the national/international sphere), and ordinary actors (non-famous professionals, or unidentified individuals); (ii) *Participant Role* this refers to the occupation/role which may be part of the identifying information included in the accompanying text, particularly in the case of elite actors (e.g. President, Prime Minister, politician); (iii) Participant Activity, which describes the kind of activity the main foregrounded actors are engaged in (e.g. walking, speaking, protesting, dancing, etc.). For a detailed coding sheet, please see https://osf.io/dc5fn.
- **Interaction:** Any semiotic mode has to be able to represent a particular social relation between the producer, the viewer, and the object represented (Kress and van Leeuwen 2006, p.43). With reference to visual communication, this translates into three strategies: *Social Distance and Shot type*, simulating social distance and its entailing psychological effects (e.g. a close-up, medium shot, or long shot); *Power Relations and Camera Angle*. In iconological traditions, relative height positions within images can relate imaginatively to cultural hierarchy and notions of power (Lutz 1993, 204); *Gaze/Eye Contact* (whether the subject establishes eye contact with the photographer/viewer, thus mediating if they are presented as a relatable subject, or an object for scrutiny); These positions are conveyed via low-angle, equal, and high-angle shots. For a detailed coding sheet, please see https://osf.io/egbnx.

**Fig. 4:**
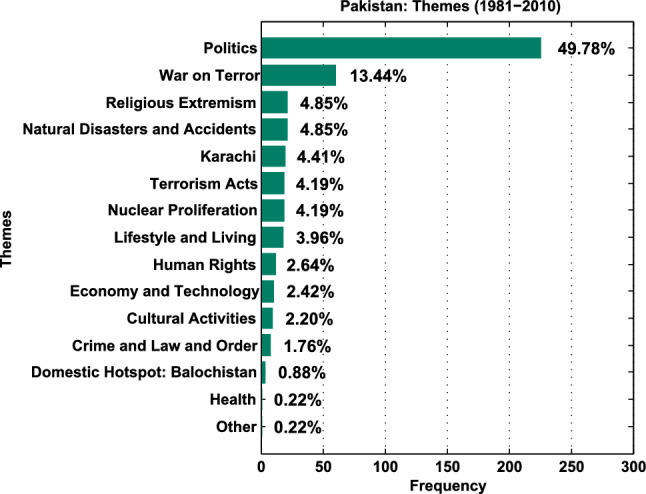
Pakistan—thematic portrayal

Mayring’s (2000) model of qualitative reliability testing was employed. The cumulative intercoder reliability score calculated using Holsti’s method of intercoder reliability is 81.23% for Representation, and 82.77% for Interaction. The data were analysed using a Microsoft Access Database.

Quantitative and qualitative results from these categories have been inductively organised into a typology of recurrent strategic narratives, using a grounded theory approach (Charmaz 2003). The broader validity of these patterns is then unpacked using a third case study, The United States, focussing on contextually relevant events surrounding the Trump Presidency. Following Bleiker’s (2019) work in using an interdisciplinary approach incorporating critical theory to analyse the role played by visuality in articulating political power, the analysis links with Butler’s (1993) notion of ‘valuable bodies’, examining how ‘othering’ in discourse leads to placing more value on certain identities, or the bodies inhabiting these identities, at the expense of others. Subjects of this kind may be seen as ‘abjected or delegitimated’ bodies which do not qualify as bodies that matter (Butler 1993, xxiv). The valuation of these bodies using language and narratives become a part of the circuits of power, an ‘ensemble of mechanisms brought into play in all the clusters of procedures used by power’ (Foucault 1980, 71). Media is one of the constellations of power apparatuses, which contribute to the construction of regimes of truth, certain reified ways of constructing and acting upon reality, reflecting “a circular relation with systems of power which produce and sustain it, and to effects of power which it induces and which extend it’ (Foucault 1980, p.132). This project focusses on a specific kind of truth producing discourse, photojournalism, and unpacks how visual cues, constructed into narratives, produce regimes of truth around what a negatively branded nation looks like, by contributing to ‘othering’ in the forms of fixed patterns of national representations. The discursive aggression inherent in such a mechanism, as Mattern (2005) articulates in her analysis of the Iraq war, in turn, has serious implications for how foreign publics perceive another nation state.

## Exploring narratives of reputational erosion: case studies

As foregrounded in the prior section, this paper uses historical data from two consistently stereotyped countries—Pakistan and Iran. In 2019, the Reputation Institute, a U.S-based data and insights company, ranked Pakistan 53 out of a list of 54 in its list of ‘the world’s most reputable countries’. Iran ranked 54 (Valet 2019). These case studies provide the basis for the heuristic typology underling the index and are complemented by a more nuanced case study—the United States, as a country that has historically both deployed, and more recently been subjected to, Negative Watch.

Viewed since the Islamic Revolution as a country ruled by ‘mad mullahs’ (Beeman 2005), Iran is often subjected to sanctions (Sen 2018) and was included, post 9/11, in President Bush’s ‘Axis of Evil’ (Miller & Sokolsky 2017). After a political détente in 2015, tensions rose again in 2020 after new sanctions (Macias & Breuninger 2020). While regularly critiqued for terrorism, nuclear weapons, sanctions, veiling and suppression of women’s rights (Roushanzamir 2004), Iran has successfully parlayed its ideology for soft power gain in Muslim countries with a Shi’ite population (Wastnidge 2015). Furthermore, Iran’s people are often portrayed favourably, while the government is depicted negatively (Delgado 2003), a narrative bifurcation reinforced by the popularity of Iran’s national cinema (Naficy 2008), the pro-democracy Green Movement (Tehranian 2008; Mortensen 2011), and the recent anti-Hijab protests (Filkins 2022). Iran’s narrative in the western media continues, consequently, to be complicated, and adversarial.

Pakistan was described by former U.S secretary of state Madeline Albright, as ‘an international migraine’ (as cited in The Economist, 2008), often perceived ‘as the world’s largest assembly line of terrorists’ (Jalal 2011, p.7), and subjected to sanctions and withdrawal of aid packages (Dawn 2012). Trust in the country was judged extremely low amongst government officials, retired military officers, business leaders, scholars, and journalists (Wike 2013). Wanta et al. (2004) note that unfavourable stories dominate the country’s coverage on television. Mughees (1995, 1997) observes that elite American newspapers favour India over Pakistan in their coverage of South Asian politics. Post 9/11, despite an alliance with United States, the elite American press maintained a critical stance towards Pakistan (Khan and Irtaza 2010). Pakistani women are often ‘othered’, embodying western fears about Islam (Hameed-ur-Rehaman 2014). Moreover, Pakistan exemplifies a narrative bifurcation opposite of Iran—post 9/11, Pakistan’s military leadership is seen more favourably than its people (Durrani and Mughees 2010; Durrani 2017). This negativity has created strong internalised pessimism within Pakistanis, spurring the desire for migration (Yousaf and Li 2015; Yousaf et al 2019).

To illustrate the validity of the typology derived from historical political analysis (Kaneva 2011), the analysis refers to other examples, in particular the United States. Traditionally viewed as a soft power giant, the US has not presented a compelling narrative to other nations since the end of the Cold War (Pamment 2014). The global sympathy generated by 9/11 was diminished by a series of military interventions (Brett & Schaefer 2019). Donald Trump’s presidency strongly accelerated reputational decline, with global publics expressing lowered trust in Trump, and American values and customs (Pew Research Centre 2017). Within the space of a year, the US went from number one to six as a place brand (Place Brand Observer 2018). The consequent erosion of global standing has opened fronts for China and Russia in their quest for global leadership (Kassab 2020); therefore, the analysis will particularly focus on the Trump Presidency, as a contextually relevant time period.

Using the proposed methodological framework, as applied to the above case studies, this study asks the following:

1. What kinds of unfavourable narratives characterise Iran and Pakistan’s story in *Time,* and how are these patterns mirrored in contemporary media discourse?
2. What are the possible implications of this process of negative characterisation?
3. What are the insights offered by this analysis towards the construction of systematic, potentially replicable systems of analysis for negative place branding?

## The Negative Watch Index (NWI): ‘Othering’ a nation brand

Building on Mattern’s (2005) idea that soft power *attraction* is socio-linguistically constructed via narratives, this paper proposes that the same is true of *repulsion*. In storytelling, a protagonist is always defined relative to an antagonist; soft power, too works dyadically. America’s Iraq military intervention relied on constructing the US as the ‘good guy’, and Iraq as the ‘bad guy’ (Mattern 2005).

Following Mattern (2005), this section takes a case study approach to investigate narrativisations of ‘Othered’ antagonists. It outlines the first dimension of the model: an index termed as the Negative Watch Index (hereafter referred to as NWI), which coheres consistent negative narratives into a typology supported by quantitative and qualitative data.

Holistically, the NWI comprises three indicators:

1. Systems of Governance
2. People
3. Thematic Portrayal

These indicators have conceptual adjacency to Nye’s indices of soft power: culture, values, and policies. Systems of governance reflect policies, and mediate values. People reflect these values in their culture, which is influenced by policy. Dominant discursive themes and perceptions of policy, culture, and values help construct the nation as a particular type of imagined community (Anderson 2006), with consequences for its soft power potential.

The following section outlines each indicator, using quantitative and qualitative data to illustrate the significance of each dimension.

## INDICATOR 1: Systems of Governance

This indicator has the following sub-dimensions: relative political stability, mode of government (democracy vs. autocracy), status (ally vs. antagonist), relative tolerance of political dissent, and economic viability.

In essence—a nation ranking high on the NWI is characterised by the following unattractive narratives: politically unstable, with an unfriendly autocratic government in charge, intolerant of dissent, and unable to guarantee the economic well-being of its citizens. Using data from Iran and Pakistan, this section demonstrates how these themes, while staple dimensions of classic patterns of image erosion, are more widely applicable to other case studies—even a soft power giant like America (Table 1).

From 2018 to 2022, the world watched in confusion as the American policy appeared to be fluctuate tweet by tweet. What President Trump said about an issue, and who he chose to work with, changed frequently. Globally, America’s traditional alliances, including those in Europe, came under increasing strain. Domestically, the Black Lives Matter Movement surged, with its heavy-handed policing eliciting strong criticism. Underlying all this were narratives of systemic economic inequality. Using data from Iran and Pakistan, this section demonstrates how these themes, while not classically associated with a soft power giant like America, are staple dimensions of classic patterns of image erosion.

*A problematic nation state is perceived as having an unreliable, unstable political system.* Iran is consistently depicted as an adversarial, autocratic orthodoxy, with persecuted dissidents. Simultaneously, Pakistan’s holistic narrative within *Time* reflects the ‘unstable system’ label. Top five recurrent Pakistani elite actors (named actors whose images appear more than five times over thirty years) include two military dictators (Generals Zia-ul-Haq and Musharraf), and three controversial, repeatedly ousted, and periodically jailed political leaders (Benazir Bhutto, Nawaz Sharif, and Asif Zardari). Alternating modes of governance and untrustworthy leaders contribute to unattractive reputational perceptions (see also Author 2020a).

Political stability is interwoven with *system of government, as valued by the ‘watcher’.* For *Time*, democracy is preferred. Autocracy, or an autocratic leader, is not. Three of Iran’s top five recurrent elite actors fall into that category (Ayatullah Khomeini, Ayatullah Khamanei, and Mahmoud Ahmedinejad). The visual coverage for all men strongly echoes Semati’s (2008) ‘mad mullah’ tropes—religious hardliners at odds with the West.

Not all autocrats are bad, though. *Political status (ally or antagonist) matters*. The autocratic ‘mad mullahs’ (Beeman 2005) are antagonists, as reflected on *Time’s* iconic covers. When Ayatullah Khomeini appears on the cover on 17 August 1987, the headline reads, ‘Iran vs. The Rest of the World’. When President Musharraf, America’s ally in the War on Terror, appeared on the cover of *Time,* the sympathetic headline reads, ‘The World’s Toughest Job—Kashmir, seething fundamentalists, political enemies—is Pakistan’s President fighting on too many fronts?’ *(*22 July 2002). Allied autocrats are also more likely to be humanised. When a smiling General Zia-ul-Haq, America’s ally in the Afghan Jihad (Talbot 1998) appears on the cover (11 March 1985) the headline asks, ‘Moving towards Democracy?’ Inside, a photo gallery showcases Zia’s personal life—playing tennis, sitting with his family, etc. Musharraf is rendered similarly relatable *(*22 July 2002)—we see images of a casually attired Musharraf walking alongside his smiling wife; ‘the first couple’ in a ‘quiet moment’. These images link with what Lowenstein (2015) observes as the western discourse praising an autocrats’ usefulness to western interests.

Significantly, the data suggest that the credibility enjoyed by an allied dictator may not extend to his people. Post 9/11, Musharraf’s images begin appearing next to those identified as extremists. For example, on 22 October 2001, a close-up of the grim-faced General is juxtaposed against a larger photo of ‘Islamic protestors’ who ‘call for jihad and Death to America’. On 12 January 2004, in an article titled, ‘Riding the Tiger’, Musharraf’s photo is placed next to one of security officials guarding an attempted assassination crime scene. A stand-out quote attributed to a U.S Foreign Policy Aide reads: ‘His survivability is very important to us. What succeeds him could only be worse’. Musharraf is part of the implied ‘Us’ here—the Pakistani people are the stereotyped ‘Other’, a frame evoking negative affect.

This narrative is underpinned by a ‘regime of truth’ (Foucault 1980, p.133) summarised as ‘Either the Autocrat or the Extremists’. The data indicate a dictator may enjoy soft power at the expense of his people. This leads to a strategically significant implication. When favoured dictators fall from grace, does this accelerate a country’s ascent along the NWI spectrum of consequences, as outlined later in the people, since ordinary people may have already been demonised, or deemed incapable of rational self-governance? Saddam Hussein, the central villain of two American led wars, was once an ally of the United States against Iran (Harris and Aid 2013).

*Governments are also judged as to how they tolerate dissent*. *Time*’s narrative valorises dissent against the Iranian regime (see also Durrani 2020). Ten Iranian men make direct eye contact with the camera, a powerful cue for establishing pseudo-social bonds with viewers (Kress and van Leeuwen 2006). Seven depict individuals critical of, and/or persecuted by, the Iranian government. For instance, a photo of journalist Raji Samaghbadi, subjected to a mock execution by the government (16 February 1981), and one of Manoucher Ganji, a dissident at risk of assassination (21 March 1994).

Finally, *news narratives judge governments according to how well they guarantee the economic progress of their people*. Thematically, stories about Economy and Technology comprise 0.78% of Iran’s data, and 2.42% of Pakistan’s data. Qualitatively, Pakistan’s visual narrative, particularly from the mid-1990s onwards, shows a widening disparity between the rich and the poor. While poverty is not operationalised and coded per se here, it may be useful for future researchers to do so, given that inequality is now a significant global issue for developed and developing countries.

While stereotypical, these governance narrative tropes are no longer limited to the routinely ‘othered’. Once, autocratic governance served as central thematic justification of American liberal humanitarian wars. More recently, ex-President Trump has openly boasted of his admiration of ‘tougher and meaner’ strongmen and autocrats, preferring a hierarchal mode of leadership for himself. He is not alone—Jair Bolsanaro (Brazil), Rodrigo Duterte (Philippines), Victor Orban (Hungary) are other similar populists who, while powerful domestically, lack international soft power (Wolf 2019). While the polysemy of China’s ‘wolf warrior’ public diplomacy allows effective management of nationalistic public opinion at the domestic level, it is less well received by global actors (Huang 2021). These are some examples illustrating the emerging algorithmically facilitated bifurcations in prioritisation of domestic vs. international soft power.

Typically, autocrats are seen as intolerant of dissent. Vivid imagery of police suppression of the Black Lives Movement (BLM) went global, typified by the iconic photo of Ieshia Evans, the unarmed African-American woman facing off against armed policeman (Bogart 2016). This instability is often accompanied by narratives of inequity—again, BLM foregrounded matters of privilege and economic inequality in the United States audiences worldwide. In essence, when the ‘watcher’ structures representations of governance about a ‘watched’ entity using regimes of truth likely to provoke negative affect amongst audiences, this leads to the discursive construction of national reputational loss.

## INDICATOR 2: People

A nation’s story is reflected strongly in stories of its people. Relevant sub-dimensions of this indicator are status and rights of children, status and rights of women, status and rights of minorities, ‘valuable bodies’ (people who are glorified and empathised with), and circumscribed roles**/**activities, often carrying connotations of violence. This section first outlines a contemporary example, then establishes the narrative typology with historical data from Iran and Pakistan, which is then linked to global trends (Table 2).

In sum, an unfavourably perceived country is characterised thus: *it treats children poorly*, *does not grant equal rights to its women*, and *persecutes its minorities*. *Citizens are seen engaging in a relatively limited variety of activities and occupations*, some of which carry connotations of violence and chaos, evoking negative affect. Finally, the choice of individuals prized within the narrative, *‘valuable bodies’* (Butler 1993) *contains implicit judgements of the relevant country’s socio-political systems*. In essence, the regimes of truth about a country placed on negative watch depict it as an entity that devalues the rights of its own people, who themselves are seen as irrational, while valuing voices that resist this status quo.

Consistent with his policy framing migrants as a security threat, President Trump infamously supported the separation of child migrants from their parents, with ‘kids in cages’ becoming a catchcall for his approach to immigration enforcement (Miroff 2020)—popularised by images of child migrants crowded behind steel fences. His visible behaviour towards women, and his ban on travellers from primarily Muslim majority countries, were viewed by international audiences with dismay, contributing to the erosion of America’s image abroad. Global audiences empathised with the activists resisting his policies. This strident, angry image of America correlated with another long simmering narrative—its fascination with gun culture. While not classically associated with America, these narratives tropes have a clear presence in the historical data set.

*Children’s rights and status* matters. A country perceived as devaluing the child an entity risks reputational loss. The figure of a child is often utilised in narratives to symbolise the collective future of the nation (Wells 2007). Pakistan’s future in *Time* appears stark (the term ‘child’ refers to a person younger than 13 years, as ascertained with the help of the accompanying text). Seven pictures feature Pakistani children exclusively. All fit two themes: the child as a future zealot (e.g. a photo of children in religious schools, identified in the caption as ‘critical in making extremists’, 24 September 2001), or the child as a victim, of war, natural disasters, crime. *Victimised minorities* also appear repeatedly in the data (Iran’s persecuted Bahai community, 20 February 1984; violence against Pakistan’s Shi’ite community, 28 September 1998).

Narratives critiquing the *status and rights of women* are similarly prevalent. Image frequency is one indicator of the relative perceived prominence/presence of woman in public spheres. For instance, from 261 images representing Iranians, 41 depict women only. The representational implication is the construction of a regime of truth about a society where woman are not ‘valued’ enough to be seen frequently enough in public spaces, or in positions of power. See Fig. 1 for a synopsis of relative frequency for both countries.

Analytically, it is interesting also to note how the designation of roles for Iranian men and women shifts across gender (see Table 2). Iranian men, viewed via the lens of *Time* over 30 years, are most likely to be politicians, religious leaders, soldiers, and activist, roles signifying power and agency. Political roles are the top category (28.57%). This is unsurprising, given *Time’s* focus on political content. Religious roles are next, a category dominated by religious leaders such as Ayatollahs Khomeni and Khamenai. Women are almost entirely absent from political roles. The concentration shifts instead to activist and professional roles (most of the data for which comes from the last decade of the sample), victims, citizen, and unclear/other categories (Table 3).

**Table 1:** A summary of NW1 Indicator 1—Systems of Governance

INDICATOR 1: SYSTEMS OF GOVERNANCE	Relative Political Stability Has an unstable, unreliable system
	Modes of Governance Aspects of Autocracy
	Status (Ally vs. Antagonist) Relationship with the ‘watcher’
	Relative Tolerance of Political Dissent Intolerant of dissent
	Economic Viability Unsuccessful facilitation of the economic progress of a people

**Table 2:** A summary of NW1 Indicator 2—People

INDICATOR 2: PEOPLE	Rights and Status of Children Treats children poorly
	Rights and Status of Women Does not grant equal rights to women
	Rights and Status of Minorities Persecutes Minorities
	Valuable Bodies Who is prized/lionised within the narrative by the watcher? Choices may contain implicit judgements of ‘watched’ country’s socio-political systems
	Roles and Activities Citizens seen as engaging in limited range of activities/occupations, often those with negative affective resonance

**Table 3:** Roles—Iranian Men and Women

List of roles (Iran)	Male		Female	
	#	%	#	%
Political Role	54	28.57	1	2.44
Religious Role	40	21.16	2	4.88
Activist Role	24	12.70	9	21.95
Military, Police, and Law Enforcement	33	17.46	3	7.32
Victims	7	3.70	6	14.63
Professional Roles	18	9.52	8	19.51
Citizen	2	1.06	7	17.07
Law Breakers	2	1.06	–-	–-
Unclear/Others	9	4.76	5	12.20
Total	189		41	

Quantitatively, the impact of female actors in roles neutrally labelled as ‘citizen’ is not sufficiently clear. However, the compositional context in which they appear is qualitatively significant, as these photographs are deployed to mediate reader perceptions of incumbent Iranian governments. Photographs featuring these unnamed women are juxtaposed alongside the image of a specific leader, and the meaning potential is circumscribed with the help of accompanying text. The photos serve as a judgement of, and reflection on, how a specific political leader is viewed from an American foreign policy perspective. For example, an article published on 8 August 1998 features a photograph of Ayatullah Khomeni, alongside with the headline,‘War and hardship in a stern land’. Two accompanying photographs show women: faceless black silhouettes in chadors, their backs to the camera, one at the cemetery, and one at a beach. With no interactional cues to connect them with readers, the depicted women are a fair reflection of the ‘stern land’ hinted at in the headline. In contrast, an article published on 19 January 1998, titled “New Day Coming?” about Iran’s reformist new President Khatami, features Khatami’s picture flanked by two images. The left side shows a smiling young woman putting on lipstick using a mirror, signifying westernisation and progress. The image on the right shows a young boy burning the American flag, representing the right-wing ideologies of old Iran. The visual metaphor places Khatami as caught between two worlds—what is and what could be, symbolised by a woman, and a child.

Notably, semiotic cues encouraging relatability and empowerment, such as direct eye contact and low angle (Kress & van Leeuwen 2006, p.17) are conferred on Iranian women who are activists and trailblazers, resisting the system. The first named Iranian woman to make eye contact with the camera (15 May 2006) is activist Shirin Ebadi (see also Durrani 2022). From 1990s onwards, these same cues are conferred on Pakistani women who are either activists (e.g. Asma Jehangir, human rights lawyer, 28 April 2003) and victims (Fakhra Khar, domestic violence victim, 20 August 2001). Here, *a ‘valuable’* body is one that symbolises either resistance or oppression—an indictment of governance and culture, which reflects unfavourably on the national brand as whole (Durrani 2022).

The Anholt-Gfk Roper City Brands Index (ACBI) defines ‘people’ as a crucial dimension of soft power—does a population have a reputation for competence, openness, friendliness, and tolerance?^1^ The stereotypical Australian lives by the beach, loves surfing, and barbecues (Esposito 2014). These stereotypes carry attractive affective connotations—Australia is ranked 8 in the Reputation Institute’s list of reputable countries (Valet 2019).

In contrast, the data reveal that Iranians and Pakistanis are *likely to be portrayed in limited roles and activities, low on attractive affect*. In all, 56.15% (*N* = 210) of Pakistani actors are portrayed in roles associated with politics, or with military/ law enforcement; 50. 97% (*N* = 133) of Iranian actors are portrayed in roles associated with politics, religion, or military/law enforcement. Further patterns are revealed when results are refined by gender, status, and specific activity type. If Australian men are compulsive surfers, ordinary Iranian men, in *Time,* are likely to protest, carry/use weapons, and get arrested/be held captive (45.25%, *N* = 43), activities with negative affective connotations. See Table 4.

**Table 4:** List of top ten activities for Iranian Men (Ordinary Actors)

Activity type	Frequency (#)
Protesting/rallying	21
Posing	17
Using/carrying weapons	16
Arrested/held captive	6
Walking	6
Speaking/gesturing	6
Injured and resting	5
Corpse	5
Reading	3
Working in a nuclear facility	2
Celebrating	2

Future research may examine the differences between the roles and activities characterising people in popular vs. unpopular national brands. Are people from ‘attractive’ nations represented more variably? Do media narratives about such nations feature more artists, scientists, or athletes, etc. roles evoking positive affect? How does this process shift for countries experiencing soft power decline?

The global prevalence of populism, which thrives on polarisation and ‘othering’, has left some nations reputationally vulnerable, in terms of NWI’s ‘people’ dimension. Radicalised voting blocs are more accepting of harsher treatment of designated ‘others’ within their countries, with their leaders prioritising short term electability gains over long-term national reputational outcomes. Incarcerating child migrants (Miroff 2020) may have eroded America’s international credibility but it fits well with Trump’s anti-migrant policies, popular with his voters. Aung San Suu Kyi’s unsympathetic stand on the genocide of minority Rohingya Muslims in Myanmar meant her international image changed from peace icon to pariah but her domestic loyalists remained steadfast (Ellis-Peterson 2015). In 2014, Jair Bolsonaro told a Brazilian female member of Congress, “I wouldn’t rape you, because you don’t deserve it”. His rhetoric targeting women, the LGBTQI community and minorities only served to strengthen his electability as President in 2018 (Assiss and Ogando 2018). Gun violence has impacted America’s international brand equity but the policies surrounding it remain firmly entrenched amongst certain voting blocs. Future research may examine: For a country’s reputation, what are the long-term consequences of leadership prioritising domestic hard power, over international soft power?

Systems of governance and stories of people are embedded within ‘big picture’ discursive themes. The final indicator examines these broad themes. Shaped by unfolding events, and source sample/platform, this dimension should be flexibly operationalised for chosen case studies.

## Indicator 3: Thematic Portrayal

Stories about a country are characterised by certain dominant narrative themes and events. In 1988, America’s victory over USSR would have dominated headlines. In 2018, Trump’s handling of Covid 19 was a critical theme impacting global perceptions of the United States during his presidency (Schaefer and Brett 2018). The 2008 Beijing Olympics foregrounded China’s cultural assets, whereas the 2019 Hong Kong protests foregrounded Beijing’s authoritarianism. Lifestyle stories are a historical staple of Australian reputational narratives—post Covid, this focus shifted to its harsh border closure policies. It is important, therefore, to get a longitudinal view of events/ themes characterising a country’s coverage, and therefore reputation, to ascertain where the balance lies, between ‘soft’ and ‘hard’ news themes.

A key source of reputational credit is ‘soft news’ (culture, technology, lifestyle etc.). Analysis indicates roughly two-thirds of the coverage for both countries comprises hard news. Holistically speaking, in terms of overall data trends and image frequency, Iran received 376 images, Pakistan, 454 images (see Fig. 2). Iran received more coverage during the 80s. Pakistan received more coverage post 9/11.

In terms of themes/events, Iran received extensive coverage during the 1980s (*N* = 237), arising from the hostage crisis, the Iran–Iraq War (Black 2010), and Operation Praying Mantis (Lamothe 2015). Consequently, 75.43% of the data focus on Politics (a theme including photos of political leaders and officials, elections, diplomats, etc.) and Military Conflicts. Coverage dips in the 1990s, resurging in the 2000s, because of the Green Movement (Tehranian 2008). In all, 12.44% of Iran’s coverage constitutes soft news (Lifestyle, Cultural activities, Health, Economy and Technology, Education), data primarily appearing in the 90s and early 2000s, depicting a softer picture of ordinary Iranians.

74.9% of Pakistan’s data focus on Politics, Military Conflict, Terrorism, Crime, and Religious Extremism. All top five categories carry negative affective resonance. Typical images for ‘politics’ include military dictators, politicians, activists, rallies, voters, evoking unstable systems of governance. ‘War on Terror’ (WoT), a post-9/11 category, is next. ‘Religious Extremism’ features conservative religious activists and leaders, sympathetic to militants, and pre-9/11 religiously inspired violence. ‘Karachi’ encapsulates the breakdown of law and order in Pakistan’s largest city. In all, only 6.38 percent of the data comprise soft news themes (Fig. 4).

This particular data set is impacted by a genre/publication constraints—*Time* is geared towards hard news. Therefore, Indicator 3 is designed to be open-ended. Contemporary media platforms and their audiences are inherently diverse, global events are always in flux, and thematic trends will vary accordingly. The central purpose here is to analyse soft power potential of key themes, as expressed, possibly, via hard vs. soft news bifurcations.

**Fig. 5:**
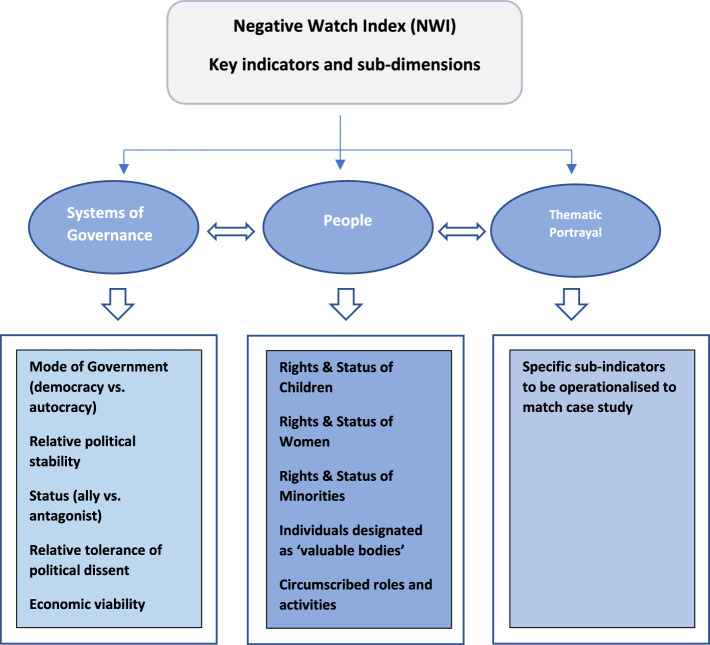
Negative Watch Index: A Synopsis

Figure 5 provides an overview of the NWI. Essentially, these are ‘commonsensical’ discourses, embedded in news narratives, with negative affective associations, connoting soft power decline.

**Fig. 6:**
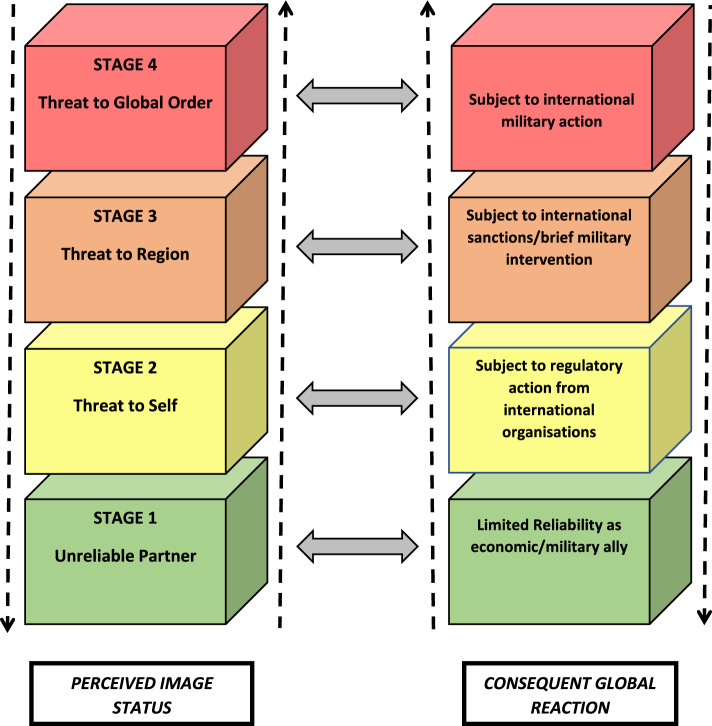
NWI: Potential Outcomes

## Implications of NWI—establishing a spectrum of effects

So, what might happen when a nation is consistently characterised with negative narratives?

Reputational security (Cull 2022) is reliant on reputational assets *and* vulnerabilities, where soft power shores it up, negative watch depletes it. Too much of the latter may impact a state’s overall security. Therefore, this section outlines a heuristic model, supported by examples, demonstrating a spectrum of possible outcomes for long-term reputational erosion. This model analytically complements the index; higher NWI scores translate to greater reputational risk levels. Concurrent application of both evaluative devices enables holistic assessment of a country’s relative reputational vulnerabilities. The following discussion outlines the model, supported by relevant historical analysis from the primary case studies.

Figure 6 illustrates an aggregated chain of events, with interlinked outcomes. The colour coding references the Defence Readiness Condition (DEFCON), an alert system used by the US Armed Forces, to better highlight increasing levels of risk from reputational erosion.

Column 1 (left) illustrates a chain of holistic, bi-directional international reputational strategic narratives: unreliable partner, threat to self, threat to region, threat to global order. Each stage is paired with consequent global reactions. The paired stages are aggregated. Unreliable partners find it hard to attract allies. This compromises a country’s capacity for effective self-management and may invite international regulatory action. Continued destabilisation may turn a country into a regional threat, inviting sanctions or a brief military intervention. Over time, sanctions and/or military strikes may induce sustained economic stress and decreased agency, thus depleting national hard and soft power reserves. Finally, complete reputational and structural collapse may leave a country vulnerable to global military action to protect the world, and to ‘rescue’ it from itself. Each stage can become a self-reinforcing short circuit (indicated by double headed arrows). For instance, a country perceived as unreliable ally has trouble attracting allies. The longer it is perceived as unreliable, the more credible that status becomes. The stages, while not strictly linear, are still interlinked and aggregated, and bi-directional (indicated by vertical arrows—the same country can have ups and downs). A stage 4 country likely travelled through other stages.

Iraq and Afghanistan are stage 4 examples. Iraq has been subjected to military action twice, in 1990 and 2003. Afghanistan was invaded in 2001, post 9/11. In both cases, to paraphrase Nye (2010a, b), it was the story that justified the launch of an army. Iraq was successfully ‘othered’ by the United States and its allies in pre-war information campaigns (Mattern 2005) with its vilified dictator, Saddam Hussein, serving as a powerful antagonistic symbol. The same was true of the Taleban. Liberal humanitarian justifications of war about brutal regimes oppressing their own people dominated pre-invasion discourse in both instances (Fahmy 2004; Klaus and Kessal 2005; Parry 2011). America, and its allies, successfully deployed the indices of ‘governance’ and ‘people’ against their adversaries.

This final stage is likely precipitated as a dyadic soft/hard power outcome. One, the ‘watched’ country is low on soft power—Saddam and Taleban were already vilified symbols. Meanwhile, the United States and its allies were highly credible (Mattern 2005). Prolonged existence at stage 3 (sanctions) inevitably damages national economy, and military prowess; therefore, Iraq was also low on hard power, with a gutted economy. A generation later, however, the protagonists of the above narratives are no longer the uncontested ‘good guys’—demonstrating that reputational credit reserves are finite, and subject to change. After its chaotic, eventual Afghanistan exit, America’s reliability as an ally came under scrutiny, a stage 1 concern (Kausikan 2021), with its involvement in multiple long-term conflicts serving to erode international good will (Brett and Schaefer 2019).

Iran, sanctioned repeatedly, currently exists between stage 2 and 3. Military invasion was considered post 9/11 (Beeman 2005), and in 2020 (Quinn 2020). However, Iran possesses substantial regional soft power (Wastnidge 2015) alongside hard power reserves, complicating its further progression along the spectrum. Sanctions on and suspension of aid to Pakistan has varied, in keeping with its strategic importance to the United States, with sanctions imposed in 1990, post Afghan Jihad, and in 1998, after it declared its status as a nuclear power. After 9/11, loans were forgiven, and aid granted (Dawn 2012). The Trump administration placed the country on the Financial Action Task Force’s (FATF) grey list, a global body that combats terrorist financing and money laundering (Rizvi 2019). The country vacillates between stage 1 & 2. Like Iran, its inherent hard power reserves likely impede further progression.

In essence, each stage of the perceived image status operates as a regime of truth (Foucault 1980), reliant on repeatedly iterated strategic narratives which serve to discursively value, and/or devalue (Butler 1990) nation states in ways that have real, material consequences for constraining an impacted nation state’s agency by modulating circuits of power (Foucault 1980). Once the presence of weapons of mass destruction was established as a regime of truth, the aggressiveness inherent in this discourse triggered a series of events forming global circuits of power, which then manifested as material aggression—war. An extended stay on negative watch depletes a nation’s credibility reserves—Iraq was, by then, perceived as a country which could not look after its people, characterised by poor governance systems—its ability to exercise agency in constructing its own narrative is minimal. A complete collapse of reputational security can, therefore, potentially create situations where a nation state’s very sovereignty at risk. It is, therefore, useful for nation states to predictively map the stages of depletion of their credibility, and therefore, agency, in the global arena.

It is important to note here that in practice, these stages manifest in complex ways, and that is possible for a nation to reset its image even after reaching stage 4—post WWII Japan and Germany are two such examples. This heuristic process is provided here to illustrate the strategic significance of mapping reputational decline by conceptualising, and tracking, the consequences of being on Negative Watch. Future scholars may develop, expand, and refine this heuristic, within relevant contextual and temporal dimensions.

If nations are imagined communities, with the media as the site where these imagined narratives are negotiated (Lems et al. 2016), then nations on Negative Watch have depleted agency to imagine, create, and disseminate their stories. Power is often a zero—sum game. This gives nations with higher reputational credit reserves a strategic advantage—the power to imagine their preferred strategic narratives onto the weakened party. It is therefore useful for practitioners to measure, anticipate, and remedy, a country’s accumulating scores on the NWI, while tracking its progression along various stages.

## Negative Watch—contemporary strategic relevance

Moving away from historical analysis, this section illustrating the contemporary strategic significance of Negative Watch. It also suggests further possible conceptual links and strategic applications within the realm of international relations, with the intent of promoting further debates.

*Firstly, Negative Watch can be deployed as a manifestation of ‘sharp power’, a tool in a process termed here as adversarial branding*. The rise of sharp power (Nye 2021) allows actors to perforate national information ecosystems, eroding a target country’s credibility amongst target publics, by promoting ‘Negative Watch’ narratives. I use the term ‘adversarial branding’ here, to differentiate this process from propaganda, a term with strong associations of falsehood. Dismissing weaponised information trends entirely as propaganda mitigates significance of phenomena ripe for generating negative place branding. In the U.S, the assault on Capitol Hill *did* take place (Mellen & Taylor 2021). Roe vs. Wade *was* overturned (Stein 2022). Data, from the chosen case studies here, are derived from credible sources. It is entirely possible to invoke Negative Watch by focussing on selective, stereotypical, fact—checked narratives—an adversarial branding process easily turbo-charged by social media algorithms.

Promoting positive stories, without understanding and countering fast spreading negative narratives, diminishes the strategic impact of soft power initiatives. For this reason, *the theory of Negative Watch is highly relevant to the realities of the networked digital era*. Digitalisation has opened the field up (Pamment 2021; Manor 2022), and this broadening needs to be a part of the theory building efforts (Manor 2019). Social media algorithms often prioritise negative, divisive content, are vulnerable to propaganda generated outside of national boundaries, and have proved devastatingly effective in inspiring vilification of individuals, communities, and countries, with speed, and at scale (Milmo 2021). This theory provides a window into conceptualising and measuring the implications of a process termed here as ‘algorithmic radicalisation’—which can be described as the way social media facilitates the creation of highly polarised political and social world views through siloed filter bubbles serving as echo chambers—for public diplomacy. As such, it fits with emerging debates which posit that Public Diplomacy no longer fits the simplicity of uni-directional models. Disruptive global events highlighting the multi-polarity of the contemporary global political order, such as COVID 19 (Manfredi-Sanchez 2022) and the Russia–Ukraine war (Kaneva, Dolea, & Manor 2023) have raised new questions about how public diplomacy and place branding do, and should, work, in a world where nations actively utilise multi-platform approaches to enhance their own credibility, while often simultaneously undermining the reputation of rival states. This trend also links with the way the appeal of Western values—a central premise of Nye’s original conceptualisations of soft power—is diminishing, while the use of anti-Western narratives as a branding by major non-Western powers such as China and Russia is on the rise. This calls for new, de-westernised ways of thinking about how public diplomacy works in the digital era, particularly in the Global South (Repnikova 2022). The West is no longer the pre-dominant ‘watcher’, with a uni-directional monopoly on how information flows through global information systems, as it did in the golden age of legacy media. By creating frameworks for interrogating and problematising mechanisms facilitating reputational erosion and power loss, this paper extends, and moves beyond, post cold war, soft power centred, western modes of theorising which focus on credibility/power gain, with credibility/power loss often discursively characterised as an issue relevant primarily to routinely ‘othered’ countries in the Global South.

To that end, this theory also links directly to emerging work in public diplomacy theorising about the role of adversarial non-state actors (Popkova 2020; Pamment 2022) in enacting target-based and issues-based disruption enacted by non-state actors (Pamment 2021)*. Deployed as a form of ideological power (Lukes 2005), Negative Watch is equally useful for offensive and defensive reputational strategies.* The NWI offers a framework for analysing these phenomena. This is particularly relevant for examining emergent trends in digital diplomacy employing cross-platform influence that replaces state media reach, as was evidenced in the Kremlin’s strategies for to circumnavigating EU sanctions on Russian state media (Pamment 2022). Let us assume Country A wants to enhance its regional/ international credibility, at the expense of Country B. It could, hypothetically, select timely negative narratives about, say, Country B’s ‘governance’, using algorithms/bots to push these, while promoting its own governance systems. An understanding of NWI would be useful to both Country A (for extending its power) and Country B (timely documentation of these narratives may predict reputational erosion). Notably, this conceptualisation echoes contemporary trends. A 2018 RAND corporation report has extensively documented Russia’s regional and international deployment of social media to discredit western powers (Bodine-Baron et al. 2018). Within the digital infosphere, the emergence of non-state actors serving as ‘alt agents’ of public diplomacy aiming to connect with foreign constituencies (Popkova 2020) represents an irreversible trend. This theory provides a framework for understanding and documenting the communication strategies employed by such alt agents.

*As an offensive reputational strategy deployed by alt agents, Negative Watch can be implemented for discrediting national images, by disrupting national identities. Again, the* theory offers a framework for analysing tactics associated with this process. Fan (2010) notes that national identity forms the basis for a national image, which then translates into a national reputation*.* Using social media, Country A can place Country B’s ‘governance’, and certain demographics of ‘people’, on Negative Watch, within Country B’s domestic infosphere. The resultant identity upheaval would disrupt national image, and therefore, reputation. The 2016 American election is an example. Conservatives were targeted with posts on immigration, race, and gun rights. Liberal activists were encouraged to stage rallies (BBC 2018). The ‘people’ element of the index split, with each placing the other, on Negative Watch. This manipulation of the infosphere, with its resultant identity polarisation, was attributed largely to America’s historic rival, Russia (Abrams 2019), and complicated global perceptions of America’s nation brand. As Abraham Lincoln famously noted, a house divided against itself, cannot stand. Undermining a national identity is a key pathway to undermining a national reputation.

## Future applications and limitations

*The suggested indictors of NWI are a useful beginning for context sensitive operationalisation.* The relevance and ultimate effectiveness of soft power depends on the perception and response of its target audience (Fan 2007). The same is true of Negative Watch. For Country A seeking to discredit Country B, culturally resonant messaging would differ for say, audiences in Middle East and Europe. Therefore, the sub-themes of each indicator may be operationalised differently, depending on the watcher, the watched, the platform, audience, and historical context. Governance narratives about the US would look different in Obama and Trump eras, varying, depending on who told the story, using what platform, and for which target audiences, and when.

*This data set is limited by its historical context*, and its focus on print media. Social media is the next logical sample source. Application of the model to further case studies where national reputation has fluctuated over time is also recommended.

Finally, as acknowledged earlier, *the loss of soft power is a complex phenomenon, and this paper limits itself to examining its communicative dimension*. While stereotypes may play a role in shaping negative reputations, these reputational deficiencies often arise from structural issues. Reversing Negative Watch is not, therefore, a simple matter of showcasing strengths. Addressing systemic issues is an established path to sustainable, long-term reputational rehabilitation. This has been true for nations once considered as international pariahs, as was the case for South Africa when it moved beyond the apartheid, Poland when it moved beyond communism, and Germany and Japan, in the aftermath of World War II. It is therefore important for policy makers to distinguish where reputational issues lie on the spectrum between adversarial branding, which may draw on genuine systemic issues and fissures, as opposed to pure disinformation. Future scholarship may therefore extend this framework interpolating other variables, beyond communication, impacting soft power loss. Future scholars may also extend this epistemology beyond place brands to create frameworks for measuring reputational loss for other kinds of brands, such as global corporate brands.

## Conclusion

This article extends Joseph Nye’s theory of soft power, proposing a new concept: Negative Watch—the systematic depletion of a country’s national reputational credit reserves through the communicative dimension, leading to a crisis of political legitimacy and credibility. To this end, this article proposes an index documenting unfavourable narratives characteristics of problematic nations, while conceptualising a spectrum of outcomes generated by reputational collapse. It then establishes the contemporary significance of this theory. In doing so, it addresses an existing imbalance in public diplomacy research, where the emphasis tends to be on gaining power, and enhancing credibility, rather than on measuring and debating the significance of its loss.

Measuring reputational loss is significant in the contemporary era. As populism ascends globally, the world is retreating into national silos. Authoritarian populist leaders vie to prove ‘strongmen’ credentials to domestic voting blocs, using hawkish nativism, conveyed via algorithmically calculated narratives, deployed within digital filter bubbles. Hard power is valorised in such domestic strategic narratives, at the expense of international soft power. Boris Johnson (Great Britain), Donald Trump (United States), Jair Bolsanaro (Brazil) are some examples of this brand of politics.

This leads to new questions for public diplomacy scholars: Where governments once aspired to soft power attributes, will leaders prioritise narratives garnering higher international NWI ratings, if it gives them greater domestic electability? What happens to international political dynamics if, hypothetically, traditional ‘smart power’ nations with high hard power assets, allow a depletion of their soft power reserves? Will new emerging populist powers in the West and elsewhere, also enact ‘soft power’ in a top down, regional fashion, like Iran (Wastnidge 2015), targeting only ideological allies, and ignoring the broader global community? Are traditional soft power giants such as the United States and United Kingdom quietly stepping on the first rungs of Negative Watch? How will that affect global power dynamics?

Theory helps us make sense of reality—to understand, highlight, and predict key trends (Brunner 2019). Researchers must have the right conceptual vocabulary to chart, measure, and predict globally significant emergent trends in public diplomacy. Nye (2008) framed soft power as the means to succeed in world politics. However, the dynamics of global politics have shifted. This paper presents an initial conceptualisation of the Negative Watch model as a means of measuring the impact of these trends around soft power loss. Designed to be flexibly quantified and operationalised, it can serve as a diagnostic, pre-emptive tool used in conjunction with soft power indexes by a range of actors. Governments, PR agencies, and think tanks may employ it to assess outside actors (allies and adversaries) for strategic decision making within the realm of international relations. It can also be used for self-assessment of national reputational credit deficit, so that the right soft power measures are deployed at the right time by experts working in the fields of public diplomacy and place branding. By conceptualising new terms and processes to better reflect changing political dynamics, this article hopes to facilitate further debates.

## Footnotes

1 The Anholt-GfK Roper City Brands Index. (http://www.simonanholt.com/Research/research-city-brand-index.aspx).
